# Genetic Analysis of Growth Traits in Polled Nellore Cattle Raised on Pasture in Tropical Region Using Bayesian Approaches

**DOI:** 10.1371/journal.pone.0075423

**Published:** 2013-09-10

**Authors:** Fernando Brito Lopes, Cláudio Ulhôa Magnabosco, Fernanda Paulini, Marcelo Corrêa da Silva, Eliane Sayuri Miyagi, Raysildo Barbosa Lôbo

**Affiliations:** 1 Embrapa Cerrados/Capes, Goiânia, Goiás, Brazil; 2 Embrapa Cerrados, Brasília, DF, Brazil; 3 Postgraduate Program in Animal Biology, Physiological Sciences Department, Institute of Biological Sciences, University of Brasília, Brasília, DF, Brazil; 4 Postgraduate Program in Animal Science, Federal University of Goiás, Goiânia, GO, Brazil; 5 Brazilian Society of Breeders and Researchers, Ribeirão Preto, São Paulo, Brazil; Wageningen UR Livestock Research, The Netherlands

## Abstract

Components of (co)variance and genetic parameters were estimated for adjusted weights at ages 120 (W120), 240 (W240), 365 (W365) and 450 (W450) days of Polled Nellore cattle raised on pasture and born between 1987 and 2010. Analyses were performed using an animal model, considering fixed effects: herd-year-season of birth and calf sex as contemporary groups and the age of cow as a covariate. Gibbs Samplers were used to estimate (co)variance components, genetic parameters and additive genetic effects, which accounted for great proportion of total variation in these traits. High direct heritability estimates for the growth traits were revealed and presented mean 0.43, 0.61, 0.72 and 0.67 for W120, W240, W365 and W450, respectively. Maternal heritabilities were 0.07 and 0.08 for W120 and W240, respectively. Direct additive genetic correlations between the weight at 120, 240, 365 and 450 days old were strong and positive. These estimates ranged from 0.68 to 0.98. Direct-maternal genetic correlations were negative for W120 and W240. The estimates ranged from −0.31 to −0.54. Estimates of maternal heritability ranged from 0.056 to 0.092 for W120 and from 0.064 to 0.096 for W240. This study showed that genetic progress is possible for the growth traits we studied, which is a novel and favorable indicator for an upcoming and promising Polled Zebu breed in Tropical regions. Maternal effects influenced the performance of weight at 120 and 240 days old. These effects should be taken into account in genetic analyses of growth traits by fitting them as a genetic or a permanent environmental effect, or even both. In general, due to a medium-high estimate of environmental (co)variance components, management and feeding conditions for Polled Nellore raised at pasture in tropical regions of Brazil needs improvement and growth performance can be enhanced.

## Introduction

Beef cattle production plays an important role in Brazilian agribusiness and is expected to increase 4.4% by 2015. In terms of beef cattle, Brazil ranks first worldwide in exportation, and is the second largest producer and the third largest food consumer in the world [1]. Zebu breeds account for more than 80% of beef-producing herds in Brazil. Significant differences in growth and production traits have been detected among ecosystems and regions of this country, probably due to the great variability in climate and management systems [2].

Growth traits are influenced both by direct additive genetic effects and by maternal effects [3]. In tropical breeds, maternal effects are often omitted during genetic evaluation due to data limitation [4]. When these effects are important, but omitted, the genetic parameters are biased upwards [5], [6] and selection-efficiency is reduced.

The use of naturally polled breeds in modern cattle breeding has become an increasing trend, both because mechanical removal of horns and injuries from fights are undesirable and also because easier and safer animal management and cowhide integrity are preferred.

In Brazil, the Polled Nellore breed has been raised separately from the common Nellore in many places and has for some time been considered as a distinct. Growth and weight data have enabled trait investigation in the common Nellore in many regions of Brazil [7]–[10] and should also be used to support Polled Nellore breeding. However, data from the polled breed is scarce and insufficient to evaluate growth traits, such as the magnitude of (co)variance components. This leads researchers to consider the need to re-estimate genetic parameters and understand the covariance structure to support the development of Polled Nellore. A fully multivariate animal modeling approach would be appropriate, supporting Polled Nellore breeding with a more refined precise method. This is of great importance, particularly considering that Polled Nellore is a promising genetic resource for animal production in Tropical regions and for food supply worldwide.

Pedigree profiles and data information in general have limitations at some level, but estimates of genetic (co)variance components and genetic parameters are strategic [4] for the progress of this breed. In this context, we carried out this study to estimate direct and maternal additive genetic (co)variance components and genetic parameters for weight at 120, 240, 365 and 450 days of age, specific for the Polled Nellore breed raised on pasture in tropical regions of Brazil.

## Materials and Methods

The data set was provided by Guaporé Pecuária Company (OB brand) together with the National Association of Breeders and Researchers (ANCP) and consisted of weight records from the years between 1987 and 2010. The herds were reared in the municipality of Pontes e Lacerda, Mato Grosso State, Brazil. The climate is characterized by a humid tropical Cerrado-Amazon transition zone with two well defined seasons (rainy and dry). Average annual precipitation is 1500 mm and the altitude is 254 m.

Weight information from each and every animal allowed estimation of the adjusted weights for 120 (W120), 240 (W240), 365 (W365) and 450 (W450) days of age. This is done because all breeding programs in Brazil function with specific selection criteria, mainly standardized weights. For estimation of W120 and W240 average pre weaning daily weight gain (*pre-*WWG) was obtained for each animal using the birth weight (BW) and the weaning weight (WW) (*pre-*WWG = WW÷BW) and the formula bellow:
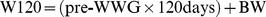


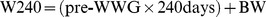



Estimation of W365 and W540 was done by obtaining the average post weaning daily weight gain (post-WWG), the latest adult weight record (AW) and using the age at the time of the last weighing (IA) (post-WWG = (AW−W240)÷(IA−W240). Estimation of W365 and W450 was done using the following formula: 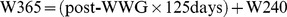


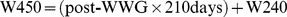



Breeding programs in Brazil do not work with average daily weight gain as selection criteria, so this trait was used only to estimate the adjusted weights at ages 120, 240, 365 and 450 days. The weaning weight (WW) and the latest adult weight record (AW) corresponds to the eighth or ninth month of age and to the seventeenth or eighteenth month of age, respectively. Based on the adjusted weights, consistency analysis was performed to exclude outliers from the data set. Any record presenting an adjusted weight smaller or greater than the average value in three standard deviation units was eliminated (Table 1).

**Table 1:** Number of records in the data, summary of statistics and significance of the source of variation.

	Trait^a^			
	W120	W240	W365	W450
Data structure				
No. Records	31,006	33,595	21,657	22,651
Summary statistics				
Mean (kg)	121.6	183.8	215.5	248.5
Standard-deviation (kg)	24.1	36.3	29.5	35.2
Coefficient of variation (%)	19.76	19.73	13.7	14.15
Effects^b^				
Sex	**	***	***	***
Herd	***	***	***	***
Year-season of birth	***	***	***	***
Cow age at calving	***	**	*ns*	*ns*

a W120, weight at 120 days old; W240, weight at 240 days old; W365, weight at 365 days old; W450, weight at 450 days old.

b indicates the significance of the source of variation;

**(P<0.01);

***(P<0.001) and *ns*, non-significant (P>0.05).

Genetic analyses were carried out by fitting a model that included the following effects: cow age as covariate; sex of the calf coded into two levels (male or female); season of birth coded into four levels (from January 1 to March 31, April 1 to June 30, July 1 to September 30, and from October 1 to December 31), years of birth and the effect of herd (seven herds).

The fixed effects included in the model were herd-year-season (HYS) of birth and sex of the calf. To define the fixed effects included in the contemporary groups, the GLM (*General Linear Model*) procedure of the *Statistical Analysis System* software [11] was used. The fixed effects that significantly influenced the growth performance were included in the subsequent model analyses. Apart from the cow age at calving (W365 and W450) other effects were significant (Table 1). Contemporary groups presenting less than three records or sires presenting less than three offspring were also removed from the final data set.

Because of the more perceptible influence of the maternal additive genetic effects and the maternal permanent factors on W120 and W240, these were not considered for W365 and W450 (Table 1). Therefore, the genetic analysis was conducted by fitting multivariate animal models. The maternal and the permanent environmental effects were not used in model 2 because these effects were not significant for W365 and W450 (Table 1). In matrix notation the mixed linear model for W120 and W240 (1) and W365 and W450 (2) was: 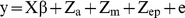
(1)

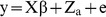
(2)


Where ***β***, represents the fixed effects vector associated with the observation (records), vector ***y*** by the known matrix ***X*** and ***a***, ***m*** and ***ep*** are the random effects vector (direct additive, maternal and permanent environmental effects) associated with records in ***y*** by the incidence matrix ***Z***; and ***e***, is the residuals vector. It is also assumed that both the systematic effects listed above and the (co)variance components included in the fitted model have a uniform a priori Gaussian distribution, whilst the conditional distributions of the direct additive, maternal, permanent environmental and residual variances present inverse Wishart distribution [12].

The marginal posterior distribution for each parameter was obtained via integration of multivariate density functions using a Gibbs sampling procedure, with a period of data collection for multi-traits of 1,500,000 iterates. The initial discard was 500,000 and the sampling interval was of 1000 iterations. Distributions (flat prior) were used to estimate (co)variance components. This was done using the MTGSAM software (*Multiple Trait using Gibbs Sampler under Animal Model*) [13]. Serial correlation and convergence for the Gibbs sampler were obtained using the GIBANAL software [14]. Convergence was monitored using the Heidelberger and Welch diagnostic tests, available in CODA library (Convergence Diagnosis and Output Analysis) and implemented in the R software [15].

The following variance components were estimated: 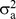, direct genetic additive variance; 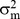, maternal additive genetic variance; 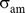, genetic covariance between direct and maternal effects; 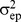, variance due to permanent environmental maternal effects; and 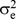, the residual variance. These components allowed the following parameters to be derived: 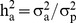, direct heritability; 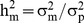, maternal heritability; 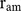, genetic correlation between the direct and maternal effect; 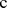, ratio between permanent environmental variance and phenotypic variance; 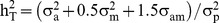, total heritability; 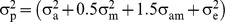, phenotypic variance [16].

At the end of all adjustments and adequacies, the data set was composed of 31.006, 33.595, 21.657 and 22.651 animals, with adjusted weights and standard age criteria of 120, 240, 365, 450 days, respectively. The animals were sons of 447, 461, 440 and 433 sires that descended from 11,771, 12,137, 9,684 and 9,705 cows, respective to the standard ages. The inverse relationship matrix was composed of 46,107 Polled Nellore individuals.

## Results

Convergence of the chain was observed running the Markov Chain diagnosis tests with 1,500,000 iterates. The posterior marginal distributions of (co)variance component estimates were accurate, tending to normal distribution (not shown). The symmetrical distributions of measures of central tendency indicated accurate analysis [17], [18].

The (co)variance matrix of maternal and additive direct genetic effects, are shown in Table 2. The estimate of residual variance was 389.64±12.74, 573.05±34.89 and 411.57±18.91, 561.91±18.13 for W120, W240, W365 and W450, respectively. The variance estimates of maternal permanent environmental effects for W120 and W240 were 12.77±1.71 and 15.87±2.44, respectively.

**Table 2:** Estimates of the mean and standard-deviation of direct (a) and maternal (m) effects of variance (on diagonal) and covariance (above diagonal) for weight at 120, 240, 365 and 450 days old.

	W120a	W240a	W365a	W450a	W120m	W240m
W120a	299.08±9.08	466.46±6.22	442.43±6.47	426.17±6.74	−52.57±5.85	−75.56±5.25
W240a		905.33±16.29	812.34±9.52	755.04±10.12	−53.37±6.01	−164.18±10.85
W365a			1075.63±12.66	1083.93±11.57	39.25±5.84	21.11±7.05
W450a				1142.78±14.13	51.84±6.16	52.44±7.38
W120m					53.30±4.27	61.60±3.95
W240m						128.37±7.62

The estimates of direct heritability and genetic correlation for W120, W240, W365 and W450 are shown in Table 3. Maternal heritability for W120 and W240 was 0.07±0.006 and 0.08±0.005 and the genetic correlation between the maternal and the direct effects was −0.42±0.031 and −0.48±0.021 for weight at 120 and 240 days old, respectively.

**Table 3:** Estimates of the mean and standard-deviation of direct heritability (diagonal) and genetic correlation (above diagonal) for weight at 120, 240, 365 and 450 days old.

	W120	W240	W365	W450
W120	0.43±0.015	0.91±0.011	0.78±0.012	0.73±0.012
W240		0.61±0.018	0.82±0.007	0.74±0.008
W365			0.72±0.011	0.98±0.001
W450				0.67±0.008

The maternal effect on weight at ages 365 and 450 days was not included in the analysis due to insignificant effect. For weight at 120 and 240 days, the maternal effects accounted for a lower proportion of total variation than the direct additive genetic effects. In general, the samples obtained for the genetic correlations (Table 3) showed no wide dispersion, i.e. the oscillations remained stable, indicating that the burn-in period considered in the analysis was reliable and allowed convergence of the chain [19]. With the aging of calves, the maternal permanent environmental effect decreased, probably because calves feed only on maternal milk until W120 (0.032±0.004), on creep feeding and some forage in W240 (0.027±0.004) and in pastures in the other categories.

The additive genetic effects accounted for a high proportion of total variation in the traits. This resulted in very high heritability estimates for growth traits, presenting upper limits of 0.49 (W120) and 0.76 (W365) (not shown). The direct additive genetic correlations between the weight at 120, 240, 365 and 450 days old were high and positive. In general, the estimates ranged from 0.68 to 0.98. The direct-maternal genetic correlations were negative for W120 and W240. The estimates ranged from −0.31 to −0.54. The estimates of maternal heritability ranged from 0.056 to 0.092 for W120 and from 0.064 to 0.096 for W240.

## Discussion

High genetic variability was observed in this herd, which was indicated by high estimates of direct additive genetic variance and heritability coefficient. Thus, it is possible to improve the traits through a selection process [20], [21], [22] aiming at genetic progress of growth traits over the years.

Strong and positive estimates of the direct additive genetic correlation between weight at 120, 240, 365 and 450 days old were observed. This indicates that improvements in one trait will imply improvements of other traits. The estimates of the direct additive genetic correlations were stronger than 0.70 at most ages and are in accordance with other studies in Brazil [23], [24] and Africa [25], [26]. With these results, it is necessary to consider the risk of selecting for greater weights at any age [27] because the later the selection is performed the greater the response is for mature weight. This is a problem because in Brazil selection is usually performed using weighing after 12 months of age, suggesting possible and undesired increases in adult size [28], [29]. Due to the strong genetic correlation estimated between these weights we suggest the selection of animals at W120 and W240.

The greatest weight gain interval is between calving and weaning [9], [30] and a clear decrease in growing rates occurs after one year of age, when the fattening and carcass finishing stages are more significant [8], [31], [32]. Following this profile, all breeding programs in Brazil are designed to consider only adjusted weights and specific standard ages.

Although the direct heritability estimates for W365 (0.72) and W450 (0.67) were greater than W120 (0.43) and W240 (0.61), selection for post weaning weights may increase the age at slaughter and the production costs throughout the years. More criteria for the selection of these traits are therefore necessary. Similar results and arguments were reported for adjusted weights at 205, 365 and 550 days in Nellore cattle in Northern Brazil [10].

Average weights for W120, W240, W365 and W450 were 121.6, 183.8, 215.5 and 248.5 kg, respectively. Average weight gain for pre-WWG and post-WWG were 0.645 kg (±0.124) and 0.288 kg (±0.162), respectively. Body weight is currently the selection criterion in Brazilian breeding programs, but if average weight gain was accounted for it would be best to select animals with greater values of W120 and W240. If correlations are strong in all ages and heritabilities are high, animal selection at the age of W120 and W240 will probably result in more precocious and heavier animals at the time of slaughter. The selection of cattle at older ages (W365 and W450) could result in animals with later growing profiles, whereas early maturing animals would maximize profits from cattle farming. The selection criteria should be based on weights, however, the decision of which weights should be chosen depends on the life period in which Polled Nellore gain more weight.

Besides the great importance of the maternal effect in extensive breeding systems in tropical regions, selection has been focused over the years on heavier weights and on the genetic additive effects. As expected [33], direct heritabilities for W120 and W240 were greater than maternal heritability. Direct-maternal correlations for W120 and W240 were strong and negative. Strong maternal effect was observed for pre-weaning growth traits, mainly for weight at 120 days, as a result of a diet restricted to maternal milk. Besides being negative, the genetic correlation between direct and maternal effects (−0.21 to −0.28) was lower than the estimates reported in other studies [34]: −0.59, [35]: −0.68, [36]: −0.50.

The genetic correlation between direct additive genetic and maternal additive genetic effects suggests genetic antagonism between both effects. Therefore, selection for direct additive genetic effects would worsen the maternal ability, making it difficult to conduct joint selection for W120 and W240 [37], [38]. This may occur when some fixed effects that influence the trait are not considered in the model [39]. However, this could also indicate greater variation between sires and dams, due to either greater genetic variance or confounding environmental effects [40]. The negative values of correlations may indicate antagonism between the effects of genes related to growth and the maternal ability, which is a major influence trait for optimal calf development [41]. However, negative correlations between direct additive genetic effects and maternal effects are frequent and often considered to be a statistical matter rather than a biological issue in animal breeding [42]–[46].

Negative regression coefficients for genetic value of daughters based on the dams phenotype were suggested by [41], [42], [47]. When negative, the covariance might make total heritability (h^2^_T_ = 0.39 for W120 and h^2^_T_ = 0.56 for W240) become smaller than the heritability for the direct additive effect (h^2^a = 0.43 for W120 and h^2^a = 0.61 for W240). This influences the expected genetic gain for the selection of W120 and W240, which is smaller and might be more appropriate. This influences the expected genetic gain for the selection of W120 and W240, which might be more appropriate as well as being smaller. However, negative values for the genetic correlation between direct additive effects and maternal effects may occur not only due to genetic antagonism, intrinsic to dam and progeny, but because of the introduction of bulls for preferential (assortative) mating and the sire x year interaction [40], [48].

Prerequisites for accurate estimates of the genetic correlation between the direct additive and maternal effects have been proposed, like the need for a consistent data set [48] that also contains records from dams and grand-dams [47]. New techniques are necessary in order to explain, illustrate and make estimates more precise [49], [50], and reach teams are exploring this area. This discussion is quite fervent among research teams.

In beef cattle farming the direct, maternal and permanent environmental effects have great importance in the pre-weaning growth rates [5], [51], [52]. In this study, the influence of the maternal effect was considered for the evaluated variables because the weight at 120 and 240 days old depends on the dams lactation length and milk yield. According to other authors [53] non-inclusion of maternal genetic and permanent environmental effects can lead to overestimation of the direct additive component and therefore, result in decreased expected genetic gain. Thus, breeding programs that consider only the direct additive genetic effects will not necessarily guarantee increased maternal ability.

This study has revealed that genetic progress is possible for the accounted traits. Maternal effects influenced the performance of weight at 120 and 240 days. Therefore, the maternal effect should be taken into account in genetic analyses of growth traits by fitting it as a genetic or a permanent environmental effect, or even both.

In general, due to the medium-high estimate of environmental (co)variance components, management and feeding conditions for Polled Nellore raised on pasture in tropical regions of Brazil need improvement so that growth performance may be improved. This will possibly reinforce the competitiveness of Polled Nellore in the meat production industry in tropical regions and boost food supply worldwide. The strong and positive estimates of direct additive genetic correlation for weight at 120, 240, 365 and 450 days old indicate that improvements in a trait should involve improvements in other traits.
